# Colchicine prevents oxidative stress-induced endothelial cell senescence via blocking NF-κB and MAPKs: implications in vascular diseases

**DOI:** 10.1186/s12950-023-00366-7

**Published:** 2023-11-24

**Authors:** Huakang Zhou, Dilaware Khan, Sajid Muhammad Hussain, Norbert Gerdes, Carsten Hagenbeck, Majeed Rana, Jan Frederick Cornelius, Sajjad Muhammad

**Affiliations:** 1 Department of Neurosurgery, Medical Faculty, University Hospital Düsseldorf, Heinrich-Heine- Universität Düsseldorf, Moorenstrasse 5, 40225 Düsseldorf, Germany; 2 Cologne Center for Genomics (CCG), University of Cologne, Weyertal 115b, 50931 Cologne, Germany; 3 Division of Cardiology, Pulmonology and Vascular Medicine, Medical Faculty and University Hospital, Heinrich-Heine-University Düsseldorf, Moorenstrasse 5, 40225 Düsseldorf, Germany; 4 Cardiovascular Reasearch Institute Düsseldorf (CARID), Medical Faculty, Heinrich-Heine- University Düsseldorf, Moorenstrasse 5, 40225 Düsseldorf, Germany; 5 Clinic for Gynecology and Obstetrics, Medical Faculty, Heinrich-Heine University Düsseldorf, 40225 Düsseldorf, Germany; 6 Department of Oral-, Maxillofacial and Facial Plastic Surgery, University Hospital Düsseldorf, Moorenstrasse 5, 40225 Düsseldorf, Germany; 7 Department of Neurosurgery, University Hospital Helsinki, Topeliuksenkatu 5, Helsinki, 00260 Finland; 8 Department of Neurosurgery, King Edward Medical University, Lahore, Pakistan

**Keywords:** Oxidative stress, Endothelial cells, Cellular senescence, Inflammation, Colchicine, NFκ-B, MAPKs, mTOR

## Abstract

**Background:**

Smoking, alcohol abuse, and hypertension are – among others, potential risk factors for cardiovascular diseases. These risk factors generate oxidative stress and cause oxidative stress-induced DNA damage, resulting in cellular senescence and senescence-associated secretory phenotype (SASP). The SASP factors in feed-forward response exacerbate inflammation and cause tissue remodeling, resulting in atherosclerotic plaque formation and rupture.

**Results:**

Colchicine inhibited ROS generation and mitigated oxidative stress-induced DNA damage. It dampened oxidative stress-induced endothelial cell senescence and improved the expression of DNA repair protein KU80 and aging marker Lamin B1. The drug attenuated the expression of senescence marker P21 at mRNA and protein levels. The pathway analysis showed that colchicine inhibited NF-κB and MAPKs pathways and subdued mTOR activation. Colchicine also attenuated mRNA expression of interleukin (IL)-1β, IL-6, IL-8, MCP-1, ICAM-1, and E-selectin. Furthermore, colchicine reduced the mRNA and protein expression of matrix metalloproteinase (MMP-2).

**Conclusion:**

In summary, colchicine blocked oxidative stress-induced senescence and SASP by inhibiting the activation of NF-κB and MAPKs pathways.

**Graphical Abstract:**

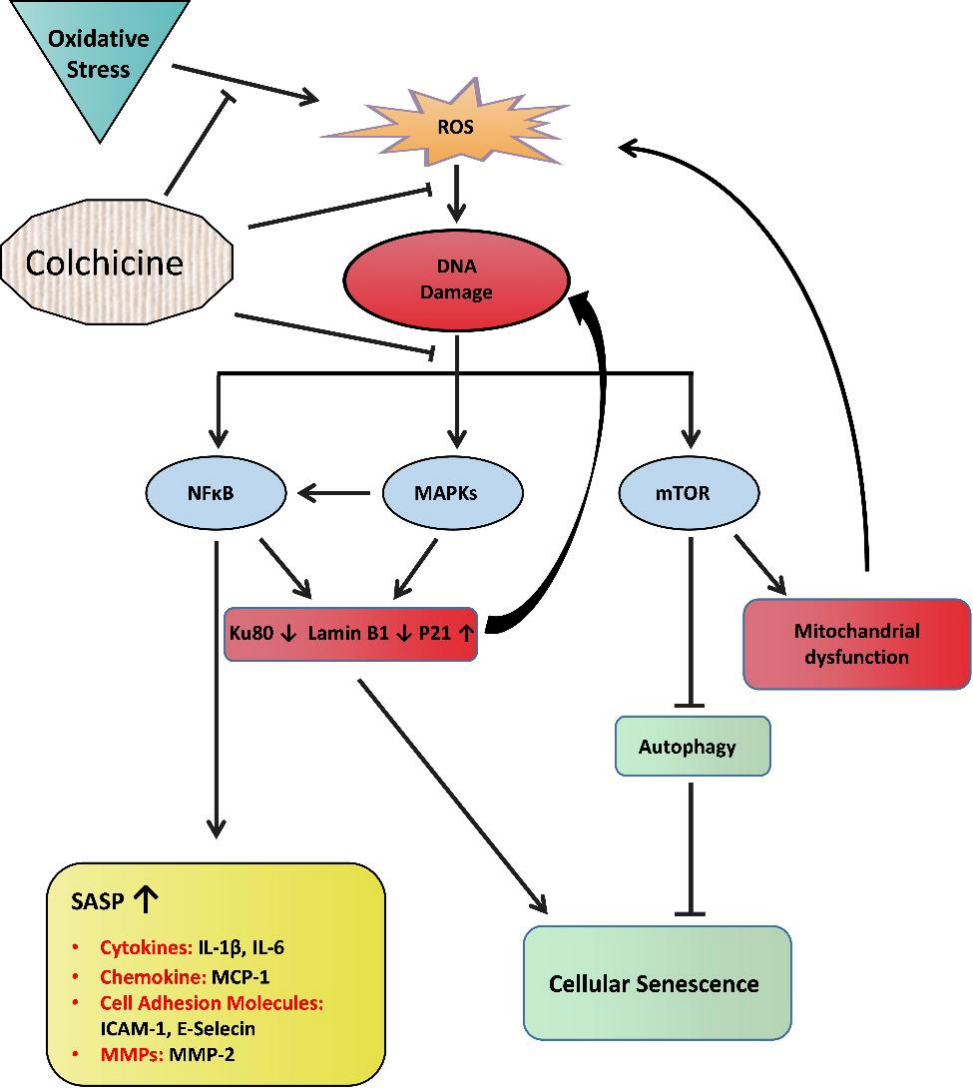

**Supplementary Information:**

The online version contains supplementary material available at 10.1186/s12950-023-00366-7.

## Background

State-of-the-art cardiovascular research suggests that cellular senescence contributes to the initiation and progression of cardiovascular diseases, including atherosclerosis, aneurysms, arterial stiffness, hypertension, and heart failure [1]. The cells can divide into a finite number, after which they become replicative senescent [2, 3]. These cells stop dividing and reach a state of growth arrest, called the Hayflick limitation [3]. In addition to replicative senescence, other stimuli, including acquired risk factors for cardiovascular diseases such as alcohol abuse and smoking, can also induce premature senescence in endothelial [4, 5] and other cell types. The phagocytic activity of monocytes and neutrophils is impaired with age, resulting in the accumulation of senescent cells in the vascular tissue. This accumulation of senescent cells can negatively affect life span and promote age-related diseases, including cardiovascular diseases [1, 6, 7]. Eliminating senescent cells improved health- and life-span and protected against cardiovascular diseases [6, 7]. Previous studies have shown the accumulation of senescent cells in atherosclerotic plaques [1, 8]. Removing these senescent cells attenuated the onset and progression of cardiovascular diseases, including atherosclerosis [1, 8].

The senescent cells acquire a pro-inflammatory phenotype known as senescence-associated secretory phenotype (SASP) [2]. These cells increase the expression and release of inflammatory cytokines, chemokines, cell adhesion molecules, and MMPs [2, 4, 5, 9]. The SASP factors released from senescent cells affect the function of the neighboring cells and induce senescence in them [10]. This paracrine process is called the bystander effect and exacerbates inflammation in the tissue surrounding senescent cells [10]. Moreover, SASP chemokines and adhesion molecules promote the recruitment of inflammatory cells into the vascular tissue [10]. The SASP factors activate smooth muscle cells and infiltrated inflammatory cells such as monocytes and neutrophils, which then release pro-inflammatory molecules and MMPs, promoting inflammation and tissue remodeling in a feed-forward response [11], thus consequently resulting in the formation and progression of atherosclerotic plaques [10]. In addition to tissue re-modulation, MMPs contribute to inflammation through their proteinase activity on inflammatory molecules [11]. The lack of these SASP factors or their inhibition by blocking their receptors has been shown to protect against vascular diseases such as atherosclerosis and cerebral aneurysms (CAs) formation and rupture in experimental animal studies [12–19].

Oxidative stress induces endothelial cell dysfunction and senescence. Cardiovascular risk factors like ethanol, smoking, and hypertension produce oxidative stress, which subsequently causes DNA damage resulting in cellular senescence. Oxidative stress and oxidative stress-induced DNA damage can activate NF-κB, MAPKs, and mTOR pathways [20]. These pathways are known to contribute to inflammation and cellular senescence [9, 20–22]. Moreover, P38 regulates SASP factors at the mRNA level through NF-κB transcriptional activity [9, 20]. Previous studies have suggested that blocking the activation of these pathways can dampen inflammation, senescence, and SASP [21, 22].

Colchicine has been used for medicinal purposes for centuries. It is an anti-inflammatory drug. Recently reported clinical trials and experimental animal studies have suggested its protective effects against cardiovascular diseases. In animal experimental studies, colchicine mitigated atherosclerosis [23, 24], thrombosis [25], cardiac damage [26], and heart failure [27]. In clinical trials, a daily dose of 0.5 mg of colchicine lowered the risks of cardiovascular events in patients [28, 29]. Interestingly, on one hand, colchicine has been reported to induce senescence in lung carcinoma epithelial cells [30] and on the other hand, it has been shown to mitigate senescence in endothelial cells exposed to ethanol [5], suggesting its contradictory role in senescence. Moreover, colchicine has cytotoxic effects. It binds to tubulin and inhibits microtubule polymerization, resulting in mitotic inhibition.

In the current study, we investigated the mechanisms through which colchicine can provide benefits against cardiovascular diseases such as atherosclerosis, thrombosis, cardiac damage, aortic aneurysms, and intracranial aneurysms. Here, we show that colchicine blocked oxidative stress-induced endothelial cell senescence and reversed an SASP via inhibiting NF-κB and MAPKs.

## Results

### Colchicine inhibited H_2_O_2_ induced ROS generation and oxidative stress-induced DNA damage

**Fig. 1:**
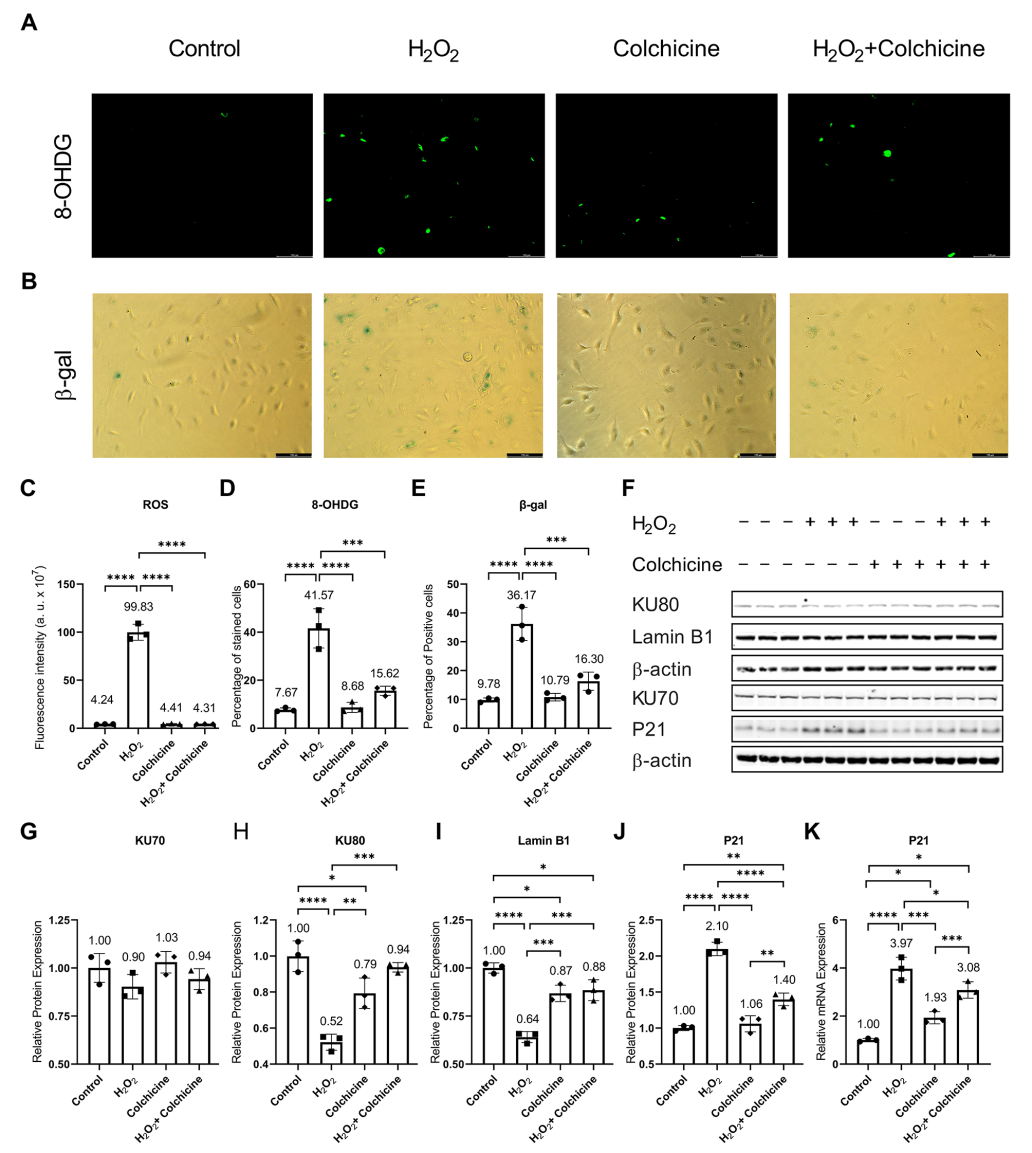
Colchicine mitigates oxidative stress and oxidative stress-induced senescence in endothelial cells. **(A)** Immunofluorescence staining for oxidative stress-induced DNA damage marker 8-OHDG, **(B)** β-gal staining. Quantification of **(C)** ROS generation, **(D)** 8-OHDG positive cells and **(E)** β-gal staining. **(F)** Western blot showing protein expression of senescence markers. Relative protein expression of **(G)** KU70, **(H)** KU80, **(I)** Lamin B1, **(J)** P21 and relative mRNA expression of **(K)** P21. β-actin was used as a loading control. The experiment was performed with biological triplicates. The data was analyzed by applying One-way ANOVA followed by Tukey’s test. Error bars represent SD, (****<0.0001, *** *p* < 0.001, ** *p* < 0.01, * *p* < 0.05), scale bar = 100 μm

To investigate the effect of colchicine on ROS generation, we treated endothelial cells with either 300 µM H_2_O_2_, 50 nM colchicine, or 300 µM H_2_O_2_ combined with 50 nM colchicine for 2 h. The endothelial cell medium alone was used for untreated control. Colchicine inhibited ROS generation in H_2_O_2_-treated endothelial cells (Control = 4.23 ± 0.21 arbitrary unit (a.u.), H_2_O_2_ = 99.83 ± 8.31 a.u., Colchicine = 4.41 ± 0.51 a.u., H_2_O_2_ + Colchicine = 4.31 ± 0.13 a.u., *n* = 3, ****p < 0.0001, Fig. 1C). Next, we performed immunofluorescence staining for oxidative stress-induced DNA damage marker 8-OHDG in endothelial cells treated with similar conditions for 2 h. Colchicine ameliorated oxidative stress-induced DNA damage in endothelial cells, a percent of 8-OHDG positive cells (Control = 7.67 ± 0.8223%, H_2_O_2_ = 41.57 ± 8.135%, Colchicine = 8.68 ± 2.109%, H_2_O_2_ + Colchicine = 15.62 ± 1.894%, *n* = 3, ***p < 0.001, ****p < 0.0001, Fig. 1A, D).

### Colchicine inhibited oxidative-stress-induced senescence

To investigate whether colchicine can attenuate oxidative stress-induced senescence in endothelial cells, we performed β-gal staining and quantified the expression of senescence markers and DNA repair proteins. Endothelial cells were treated with 300 µM H_2_O_2_, 50 nM colchicine, or 300 µM H_2_O_2_ combined with 50 nM colchicine for 24 h. Untreated cells were used as control. Colchicine obviated oxidative stress-induced senescence in endothelial cells, a percentage of β-gal positive cells (Control = 9.78 ± 0.74%, H_2_O_2_ = 36.17 ± 5.74%, Colchicine = 10.79 ± 1.31%, H_2_O_2_ + Colchicine = 16.30 ± 3.17%, *n* = 3, *** *p* < 0.001, **** *p* < 0.0001, Fig. 1B, E). Oxidative stress, colchicine, or oxidative stress combined with colchicine did not alter the relative protein expression of DNA repair protein KU70 (Fig. 1F, G, and Table 1). Both oxidative stress and colchicine reduced the relative protein expression of DNA repair protein KU80 and Lamin B1 (Fig. 1F, H, I, and Table 1). However, the relative protein expression of KU80 and Lamin B1 was significantly higher in endothelial cells treated with colchicine or colchicine combined with H_2_O_2_ than in the endothelial cells treated with H_2_O_2_ alone for 24 h. Moreover, colchicine ameliorated the relative protein expression of senescent marker P21 compared to H_2_O_2_-treated endothelial cells (Fig. 1F, J, and Table 1). The quantitative analysis of P21 mRNA expression showed that P21 expression is regulated at the mRNA level (Fig. 1K; Table 2).

**Table 1:** Quantification of relative protein expression of senescence markers, MMP-2, and proteins of NF-κB, MAPKs, and mTOR pathways One way-ANOVA followed by Tukey’s test

Protein	Treatment duration	Control	H_2_O_2_	Colchicine	H_2_O_2_ + Colchicine	*P* value
KU70	24 h	1.00 ± 0.08	0.90 ± 0.06	1.03 ± 0.06	0.94 ± 0.05	0.1359
KU80	24 h	1.00 ± 0.08	0.52 ± 0.04	0.79 ± 0.08	0.94 ± 0.02	0.0001
Lamin B1	24 h	1.00 ± 0.03	0.64 ± 0.03	0.87 ± 0.04	0.88 ± 0.05	0.0001
P21	24 h	1.00 ± 0.04	2.10 ± 0.09	1.06 ± 0.11	1.40 ± 0.09	0.0001
P65	30 min	1.00 ± 0.05	0.91 ± 0.03	1.01 ± 0.03	1.02 ± 0.05	0.0360
P65	2 h	1.00 ± 0.11	0.86 ± 0.02	1.01 ± 0.06	1.03 ± 0.02	0.0371
p-P65	30 min	1.00 ± 0.02	2.43 ± 0.41	0.88 ± 0.02	1.44 ± 0.22	0.0001
p-p65	2 h	1.00 ± 0.22	1.05 ± 0.08	1.04 ± 0.13	1.28 ± 0.05	0.1208
p-p65/P65	30 min	1.00 ± 0.04	2.67 ± 0.36	0.87 ± 0.02	1.41 ± 0.15	0.0001
p-p65/P65	2 h	1.00 ± 0.10	1.22 ± 0.10	1.03 ± 0.06	1.24 ± 0.03	0.0098
p-P38	30 min	1.00 ± 0.10	4.42 ± 0.87	0.80 ± 0.17	1.65 ± 0.12	0.0001
p-P38	2 h	1.00 ± 0.07	1.86 ± 0.23	1.21 ± 0.18	1.22 ± 0.10	0.0009
p-ERK	30 min	1.00 ± 0.13	3.28 ± 0.31	0.67 ± 0.11	1.05 ± 0.15	0.0001
p-ERK	2 h	1.00 ± 0.17	3.28 ± 0.12	1.02 ± 0.20	0.94 ± 0.18	0.0001
p-JNK	30 min	1.00 ± 0.02	1.01 ± 0.03	1.01 ± 0.02	1.03 ± 0.02	0.3316
p-JNK	2 h	1.00 ± 0.03	0.94 ± 0.02	0.90 ± 0.04	0.91 ± 0.02	0.0090
p-AKT	30 min	1.00 ± 0.08	2.55 ± 0.36	0.79 ± 0.02	1.38 ± 0.10	0.0001
p-AKT	2 h	1.00 ± 0.06	1.85 ± 0.06	1.04 ± 0.11	1.03 ± 0.13	0.0001
p-mTOR	30 min	1.00 ± 0.10	1.30 ± 0.4	1.22 ± 0.14	1.16 ± 0.19	0.1126
p-mTOR	2 h	1.00 ± 0.13	1.44 ± 0.10	1.37 ± 0.09	1.28 ± 0.12	0.0057
p-mTOR	24 h	1.00 ± 0.18	1.45 ± 0.07	1.32 ± 0.01	1.11 ± 0.14	0.0058
p-4EBP1	30 min	1.00 ± 0.17	0.96 ± 0.11	1.26 ± 0.08	1.57 ± 0.16	0.0020
p-4EBP1	2 h	1.00 ± 0.01	0.77 ± 0.05	0.99 ± 0.07	0.97 ± 0.03	0.0009
p-4EBP1	24 h	1.00 ± 0.18	0.87 ± 0.01	0.80 ± 0.06	1.10 ± 0.12	0.0547
p-S6	30 min	1.00 ± 0.01	0.94 ± 0.02	1.02 ± 0.02	1.03 ± 0.03	0.0063
p-S6	2 h	1.00 ± 0.08	1.40 ± 0.04	1.32 ± 0.07	1.26 ± 0.07	0.0003
p-S6	24 h	1.00 ± 0.02	0.94 ± 0.06	1.04 ± 0.04	1.05 ± 0.08	0.1575
MMP-2	24 h	1.00 ± 0.02	1.91 ± 0.02	1.19 ± 0.01	1.53 ± 0.10	0.0001

**Table 2:** Quantification of relative mRNA expression of senescence marker and SASP factors in endothelial cells treated with different conditions for 24 h One-way ANOVA followed by Tukey’s test

Gene	Control	H_2_O_2_	Colchicine	H_2_O_2_ + Colchicine	*P* value
P21	1.00 ± 0.06	3.97 ± 0.47	1.93 ± 0.25	3.08 ± 0.34	0.0001
IL-1β	1.10 ± 0.56	2.03 ± 0.10	0.40 ± 0.13	1.15 ± 0.31	0.0024
IL-6	1.00 ± 0.12	2.31 ± 0.28	1.74 ± 0.22	1.67 ± 0.10	0.0003
IL-8	1.01 ± 0.22	1.37 ± 0.22	1.46 ± 0.18	0.73 ± 0.02	0.0039
MCP-1	1.05 ± 0.36	1.87 ± 0.15	1.13 ± 0.19	0.38 ± 0.13	0.0003
ICAM-1	1.02 ± 0.26	2.26 ± 0.31	1.55 ± 0.06	1.54 ± 0.19	0.0012
VCAM-1	1.01 ± 0.18	1.30 ± 0.15	0.72 ± 0.08	1.42 ± 0.11	0.0009
E-Selectin	1.03 ± 0.31	2.07 ± 0.09	0.56 ± 0.06	0.68 ± 0.27	0.0001
MMP-1	1.00 ± 0.04	2.07 ± 0.07	2.81 ± 0.06	3.75 ± 0.06	0.0001
MMP-2	1.00 ± 0.07	1.34 ± 0.07	0.95 ± 0.06	0.82 ± 0.05	0.0001
MMP-8	1.07 ± 0.44	1.66 ± 0.80	1.45 ± 0.30	1.41 ± 0.11	0.5538
MMP-11	1.00 ± 0.13	1.66 ± 0.19	1.05 ± 0.08	1.08 ± 0.18	0.0020
TIMP-1	1.03 ± 0.30	0.90 ± 0.10	1.79 ± 0.33	0.72 ± 0.14	0.0026
TIMP-2	1.00 ± 0.08	1.47 ± 0.24	1.69 ± 0.20	1.58 ± 0.10	0.0056

### Pathway analysis

**Fig. 2:**
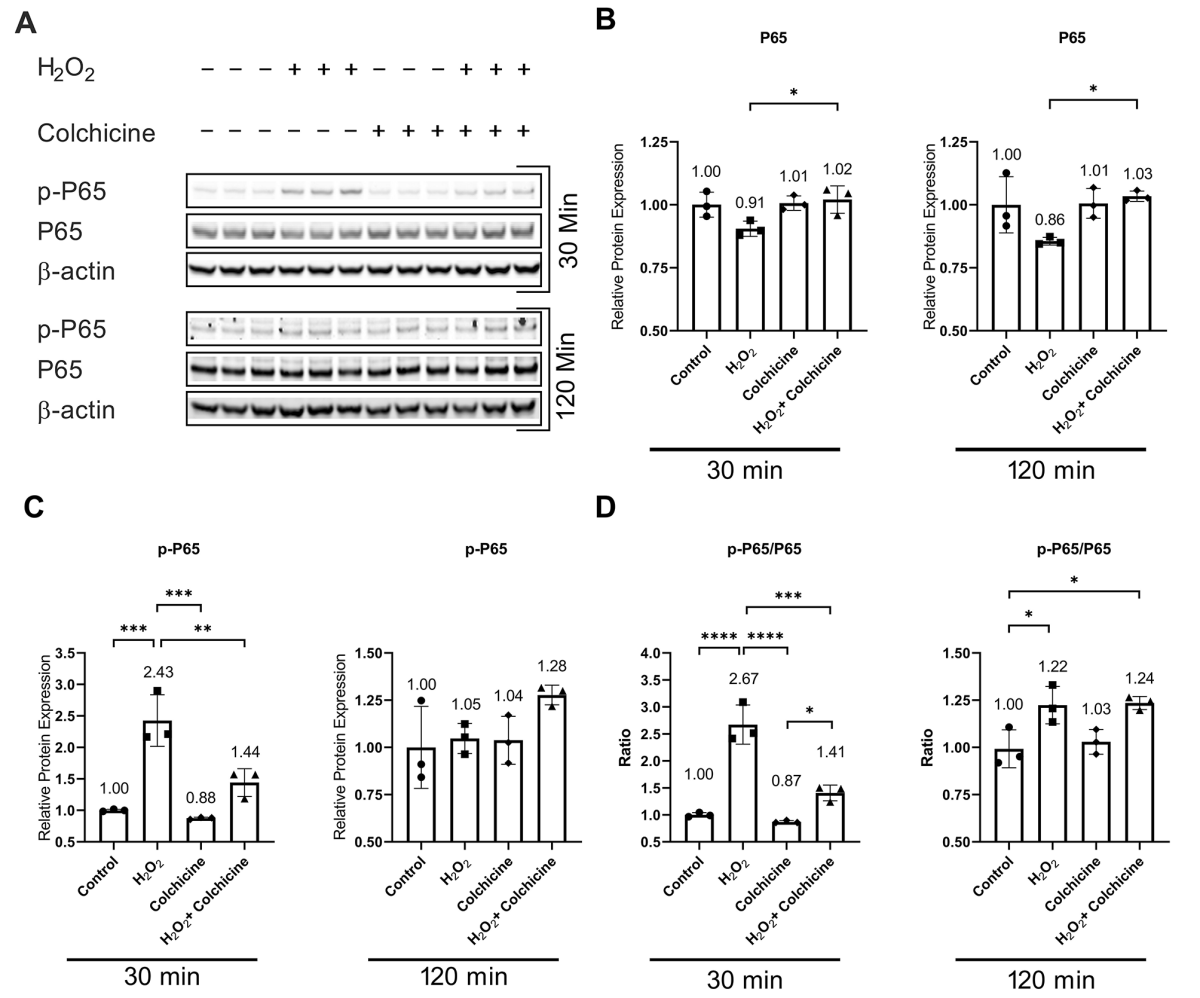
Colchicine inhibits NF-κB activation: **(A)** Western blot showing protein expression of NF-κB subunits. Relative protein expression of **(B)** P65 and **(C)** p-P65. **(D)** Ratio of p-P65/P65. β-actin was used as a loading control. The experiment was performed with biological triplicates. The data was analyzed by applying One-way ANOVA followed by Tukey’s test. Error bars represent the SD (*** *p* < 0.001, ** *p* < 0.01, * *p* < 0.05)

Because the pathways can activate and inactivate at different time points after the treatment, as evidenced by the activation and inactivation of NF-κB 30 min and 2 h after H_2_O_2_ treatment respectively (Fig. 2), therefore, we investigated the activation of NF-κB, MAPKs, and mTOR pathways at different time points after H_2_O_2_ treatment. The protein analysis showed that oxidative stress and colchicine, neither alone nor in combination, changed the relative protein expression of NF-κB subunit P65 (Fig. 2A, B; Table 1). Colchicine inhibited activation of NF-κB in endothelial cells exposed to H_2_O_2_ for 30 min and at the same time point p-P65/P65 ratio was significantly reduced by colchicine in H_2_O_2_-treated endothelial cells (Fig. 2C, D; Table 1). Neither of the treatments altered the relative protein expression of NF-κB subunit p-P65 after 2 h (Fig. 2C; Table 1). The colchicine did not decrease the p-P65/P65 ratio in endothelial cells treated with oxidative stress for 2 h (Fig. 2D; Table 1).

**Fig. 3:**
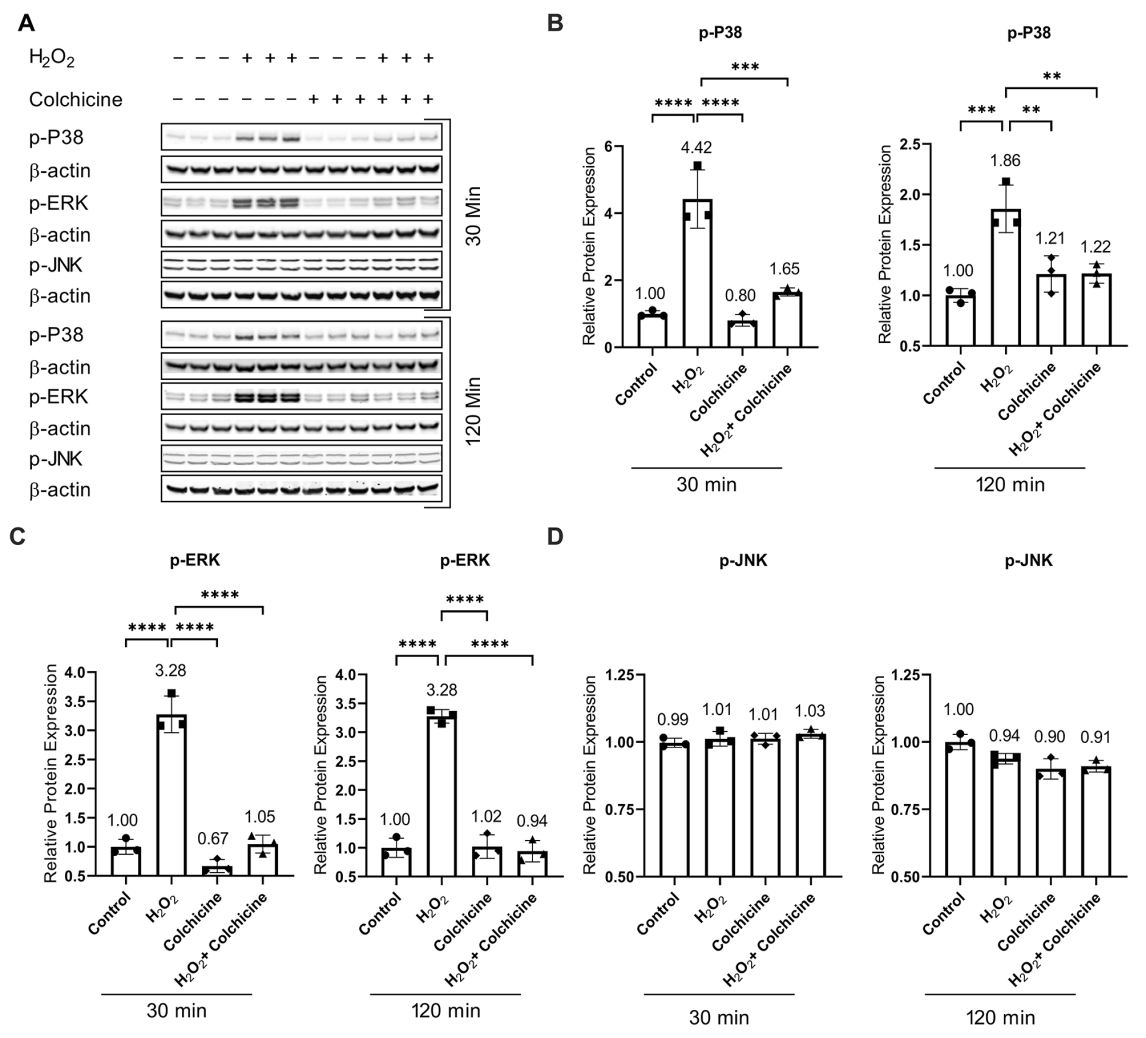
Colchicine inhibits oxidative stress-induced P38 and ERK activation: **(A)** Western blot showing protein expression of MAPKs. Relative protein expression of **(B)** p-P38, **(C)** p-ERK and **(D)** p-JNK. β-actin was used as a loading control. The experiment was performed with biological triplicates. The data was analyzed by applying One-way ANOVA followed by Tukey’s test. Error bars represent the SD, ****p < 0.0001, *** *p* < 0.001, ** *p* < 0.01, and * *p* < 0.05

Oxidative-stress-induced DNA damage can activate MAPKs [20]. Protein analysis showed that H_2_O_2_ induced oxidative-stress-activated MAPKs (Fig. 3; Table 1). Colchicine inhibited oxidative-stress-induced activation of P38 and ERK in endothelial cells (Fig. 3B, C, and Table 1). Both oxidative stress and colchicine, neither alone nor in combination, had an effect on the activation of JNK (Fig. 3D; Table 1).

**Fig. 4:**
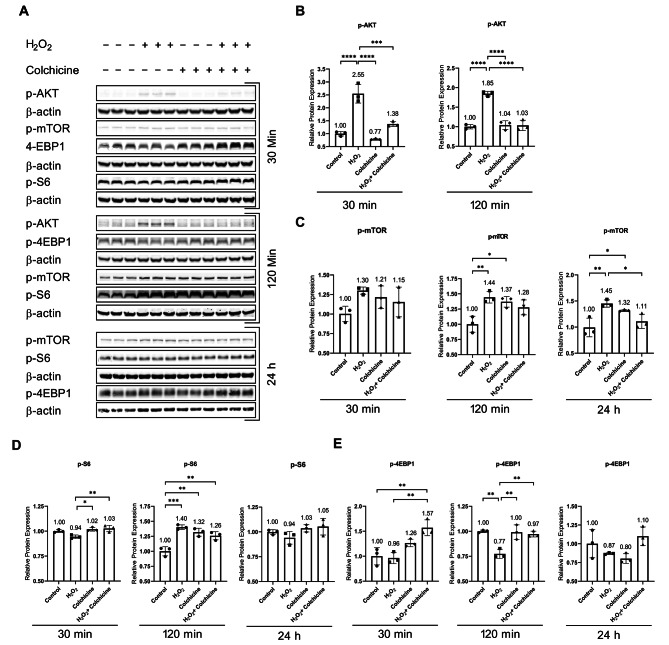
Colchicine modulates mTOR pathway activation: **(A)**. Western blot showing protein expression of p-AKT, p-mTOR, p-4EBP1, and P-S6. Relative protein expression of **(B)** p-AKT, **(C)** p-mTOR, **(D)** p-S6 and **(E)** p-4EBP1. β-actin was used as a loading control. The experiment was performed with biological triplicates. The data was analyzed by applying One-way ANOVA followed by Tukey’s test. Error bars represent the SD, (*** *p* < 0.001, ** *p* < 0.01, and * *p* < 0.05)

Protein analysis showed that colchicine inhibited oxidative stress-induced activation of AKT (Fig. 4A, B, and Table 1). Oxidative stress and colchicine, neither alone nor in combination, affected the relative protein expression of p-mTOR in endothelial cells treated for 30 min (Fig. 4A, C, and Table 1). Oxidative stress increased the relative protein expression of p-mTOR in endothelial cells exposed to H_2_O_2_ for 2 and 24 h (Fig. 4A, C; Table 1). Colchicine inhibited the relative protein expression of p-mTOR in endothelial cells treated with oxidative stress for 24 h (Fig. 4A, C; Table 1). Both oxidative stress and Colchicine, neither alone nor in combination, alter the relative protein expression of p-S6 after 30 min and 24 h treatments compared to untreated control (Fig. 4A, D; Table 1). Colchicine did not affect oxidative stress-induced relative protein expression of p-S6 in endothelial cells treated with H_2_O_2_ for 2 h (Fig. 4A, D, and Table 1). The 30-minute combined treatment of oxidative stress and colchicine increased the relative protein expression of p-4-EBP1 than untreated control and oxidative stress-treated cells (Fig. 4A, E; Table 1). Rapamycin inhibition of the mTOR pathway in most cell types inhibits mTOR and its downstream signaling molecule S6 without affecting mTOR downstream signaling molecule 4-EBP-1 [31]. Here, unlike rapamycin inhibition of the mTOR pathway, colchicine recovered the relative protein expression of p-4EBP-1 in endothelial cells treated with oxidative stress for 2 h and showed a trend of its increase after 24 h (Fig. 4A, E; Table 1).

### Colchicine ameliorated oxidative stress-induced SASP in endothelial cells

**Fig. 5:**
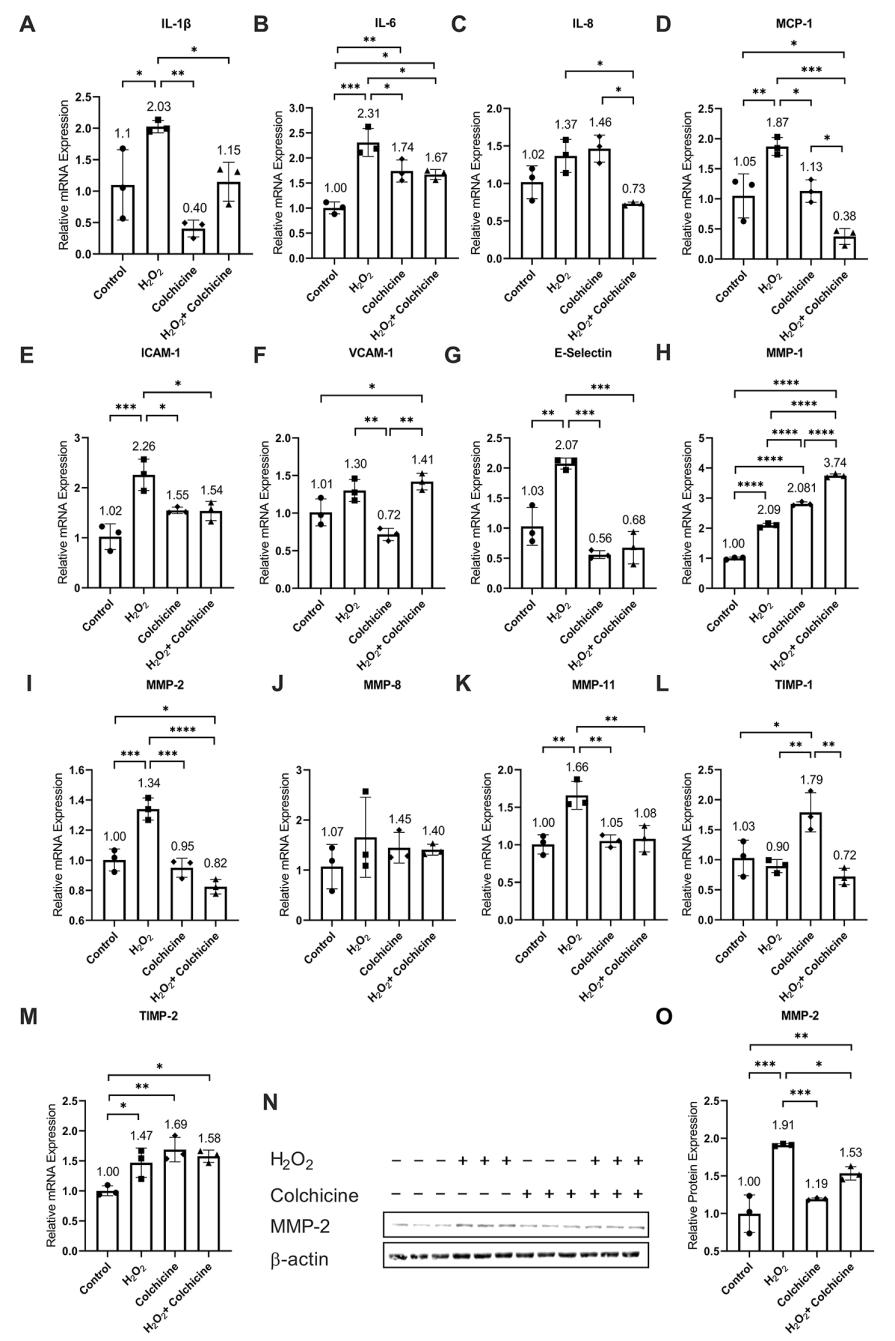
Colchicine mitigates the relative mRNA expression of SASP factors. Relative mRNA expression of **(A)** IL-1β, **(B)** IL-6, **(C)** IL-8, **(D)** MCP-1, **(E)** ICAM-1, **(F)** VCAM-1 **(G)** E-Selectin, **(H)** MMP-1, **(I)** MMP-2 **(J)** MMP-8, **(K)** MMP-11, **(L)** TIMP-1 and **(M)** TIMP-2. **(N)** Western blot showing protein expression of MMP-2. **(O)** Relative protein expression of MMP-2. β-actin was used as a loading control. qPCR data are the mean of three independent technical replicates and WB data are the mean of the biological triplicates. The data was analyzed by applying One-way ANOVA followed by Tukey’s test. Error bars represent the SD, *** *p* < 0.001, ** *p* < 0.01, and * *p* < 0.05)

Senescent cells increase the expression of inflammatory cytokines, chemokines, and cell adhesion molecules [2, 4, 5]. To investigate the impact of colchicine on SASP factors in H_2_O_2_-treated endothelial cells, the mRNA expression of SASP factors was investigated in endothelial cells treated with different conditions for 24 h. Our results showed that oxidative stress increased the relative mRNA expression of IL-1β, IL-6, MCP-1, ICAM-1, and E-Selectin (Fig. 5A, B, D, E, G, and Table 2). Colchicine averted oxidative-stress-induced relative mRNA expression of cytokines: IL-1β and IL-6 (Fig. 5A, B; Table 2), chemokine: MCP-1 (Fig. 5D; Table 2), and cell adhesion molecules: ICAM-1 and E- selectin (Fig. 5E, G; Table 2). The relative mRNA expression of IL-8 was significantly lower in endothelial cells treated with oxidative stress combined with colchicine than in endothelial cells treated with oxidative stress or colchicine alone (Fig. 5C; Table 2). The mRNA expression of VCAM-1 was significantly higher in endothelial cells treated with oxidative stress and oxidative stress combined with colchicine than untreated controls and colchicine (Fig. 5F; Table 2).

In addition to inflammatory markers, senescent cells increase the expression and release of MMPs [4, 5, 10], which consequently contributes to the initiation and progression of cardiovascular diseases [10, 32]. We performed qPCR to investigate the effects of colchicine on the relative mRNA expression of MMPs in endothelial cells exposed to H_2_O_2_ for 24 h. H_2_O_2_, colchicine, and H_2_O_2_ combined with colchicine increased the relative mRNA expression of MMP-1 and TIMP-2 (Fig. 5A, M, and Table 2). Colchicine reduced the relative mRNA expression of MMP-2, MMP-11, and TIMP-1 (Fig. 5I, K, L; Table 2). Both H_2_O_2_ and colchicine, neither alone nor in combination, affected the mRNA expression of MMP-8 (Fig. 5J; Table 2). Colchicine decreased the relative protein expression of MMP-2 in endothelial cells treated with H_2_O_2_ (Fig. 5N, O, and Table 1).

## Discussion

Endothelial cell dysfunction and senescence are known to contribute to cardiovascular diseases [1]. These cells increase the expression and release of pro-inflammatory molecules and MMPs [4, 5], which have a causal relationship with cardiovascular diseases, including atherosclerosis, thrombosis, aneurysm pathophysiology, stroke, and heart infarct [10, 32]. In the current study, we used colchicine to ameliorate oxidative stress-induced dysfunction and senescence in endothelial cells.

Colchicine mitigated oxidative stress in endothelial cells treated with H_2_O_2_ (Fig. 1C), agreeing with the previously reported findings [25, 33, 34]. Oxidative stress can cause DNA damage, growth arrest, and premature cellular senescence [2]. Colchicine attenuated oxidative stress-induced DNA damage (Fig. 1A, D). Colchicine has been reported to reduce oxidative stress-induced DNA damage in ethanol-treated endothelial cells [5] and to provide anti-oxidative effects in endothelial cells and platelets [25, 34]. The oxidative stress and oxidative stress-induced DNA damage can activate NF-κB, MAPKs, and mTOR pathways and can cause cellular senescence [9, 20]. β-gal staining revealed that colchicine dampened senescence in endothelial cells treated with oxidative stress (Fig. 1B, E). Previous studies have shown the accumulation of senescent cells in atherosclerotic lesions [1, 8] and colchicine, by inhibiting cellular senescence (Fig. 1), can prevent the progression of atherosclerosis and promote atherosclerotic plaque stability [1, 8, 10]. Moreover, colchicine improved the relative protein expression of DNA repair protein KU80 (Fig. 1H; Table 1), aging marker Lamin B1 (Fig. 1I; Table 1), and attenuated the relative protein and mRNA expression of P21 (Fig. 1J, K; Tables 1 and 2) in oxidative stress treated endothelial cells. KU80 forms a heterodimer with KU70 and repairs double-strand DNA breaks through non-homologous end joining [35]. Reduced levels of KU80 have been observed in senescent cells [36] and impaired KU80 protein expression has been shown to cause telomere shortening [37], which can consequently result in cellular senescence. The aging marker Lamin B1 maintains nuclear stability and reduced Lamin B1 protein levels resulted in misregulated non-homologous end joining and homologous repair of DNA, leading to persistent DNA damage [38]. Lamin B1 protein level is decreased in senescent cells [39, 40] due to its reduced stability [39]. Loss of Lamin B1 protein expression can cause premature senescence [40]. P21 is an inhibitor of the cyclin-dependent kinase (CDK) and establishes indefinite growth arrest of senescent cells [2]. The induction of P21 protein expression led to senescence in human HT1080 fibrosarcoma cells [41]. Though colchicine rescued endothelial cells from oxidative stress-induced senescence and improved the expression of senescence markers in endothelial cells exposed to oxidative stress, however, colchicine alone increased the relative mRNA expression of P21 (Fig. 1K) and reduced the relative protein expression of KU80 (Fig. 1H) and Lamin B1 (Fig. 1I), suggesting its DNA-damaging and pro-senescent effects in untreated endothelial cells. It is worth noting that previously colchicine has been shown to induce senescence in lung cancer cells [30]. These findings suggest that colchicine inhibited oxidative stress-induced senescence (Fig. 1B, E) by improving KU80 (Fig. 1H) and Lamin B1 (Fig. 1I) protein expression and ameliorating P21 (Fig. 1J, K) expression at mRNA and protein levels.

The expression of senescence markers and DNA repair proteins is regulated by NF-κB, MAPKs, and mTOR pathways, and these pathways are known to contribute to cellular senescence [2, 9, 20]. Protein analysis showed that colchicine inhibited activation of NF-κB, MAPKs, and mTOR (Figs. 2, 3 and 4, and Table 1). NF-κB is known to promote inflammation and contribute to cellular senescence in vitro and in vivo [9, 10, 42]. NF-κB activation impairs KU80 protein expression, subsequently resulting in telomere shortening [43], which can lead to cellular senescence. In response to DNA damage, NF-κB increased the expression of P21 [44], leading to cell cycle arrest and senescence [2, 42]. This suggests that colchicine, via blocking NF-κB activation, can suppress P21 expression (Fig. 1J, K) and improve KU80 expression (Fig. 1H), resulting in reduced endothelial cell senescence (Fig. 1B, E). Moreover, NF-κB regulates SASP responses in dysfunctional and senescent endothelial cells [9, 10], where the transcriptional activity of NF-κB in senescent cells is regulated by MAPKs [20]. MAPKs also regulate the protein expression of cell cycle arrest proteins [20]. P38 increases the expression, stabilization, and promoter activity of P53, resulting in increased P21 protein expression [45, 46]. P38 increases the cytoplasmic accumulation of HuR via phosphorylating it [47]. HuR binds to P21 mRNA and increases its stability, consequently increasing P21 protein expression [47]. Blocking senescent signals via inhibiting P38 can mitigate Lamin B1 loss [39]. ERK also promotes the transcription of P21 [20]. Previous studies have shown that inhibiting P38 activation can subdue cellular senescence [48]. Taken together, it seems very likely that colchicine via inhibiting P38 (Fig. 3B) can improve Lamin B1 protein expression (Fig. 1I) and by blocking P38 (Fig. 3B) and ERK (Fig. 3C) pathways can subdue P21 mRNA and protein expression (Fig. 1J, K) in oxidative stress treated endothelial cells. Colchicine reduced the activation of the mTOR pathway in oxidative stress-induced endothelial cells (Fig. 4C). Still, when it was administered alone, it also increased the activation of the mTOR (Fig. 4C) pathway and its downstream molecule S6 (Fig. 4D), suggesting its pro-senescent role and adverse effects on autophagy. mTOR pathway is known to play a role in aging and senescence, and its inhibition increases longevity and delays senescence [21]. The activation of the mTOR pathway negatively regulates autophagy, resulting in accumulated damaged proteins and organelles, which can lead to the progression of cellular senescence [22, 49]. Blocking mTOR activation can improve mitochondrial function and reduce ROS levels [22], which can consequently provide protection against cellular senescence.

These findings indicate that colchicine inhibited oxidative stress-induced senescence (Fig. 1) via inhibiting the activation of NF-κB and MAPKs (Figs. 2 and 3; Table 1). Because the activation of these pathways in senescent cells regulates the expression of SASP factors [2, 9, 10, 20], therefore, we investigated the effect of colchicine on the regulation of mRNA expression of SASP factors in endothelial cells treated with oxidative stress. Agreeing with previously reported findings [5], our study showed that colchicine reduced the relative mRNA expression of SASP factors (Fig. 5). The expression of these SASP factors is regulated by P38 through the transcriptional activity of NF-κB [20]. The data suggest that via inhibiting the activation of NF-κB (Fig. 2) and P38 (Fig. 3), colchicine suppressed oxidative stress-induced mRNA expression of SASP factors (Fig. 5) in endothelial cells.

Senescent cells by increasing the expression of SASP factors (Fig. 5) [4, 5, 10], contribute to cardiovascular diseases via multiple mechanisms [10]. The enhanced expression and release of cytokines, chemokines, and cell adhesion proteins by these senescent endothelial cells causes tissue infiltration of monocytes, neutrophils, and platelets. The infiltration, accumulation, and activation of neutrophils, monocytes, and platelets contribute heavily to atherosclerosis and thrombosis [10]. Colchicine reduced the activation of monocytes [24] and hindered the adhesion of monocytes to endothelial cells by suppressing the expression of VCAM-1 and ICAM-1 [25]. In experimental animal studies, colchicine dampened the infiltration and recruitment of neutrophils and monocytes into the atherosclerotic plaques [23, 24] and infarct area of myocardium after myocardial ischemia [26]. Colchicine in vitro and in vivo ameliorated platelet activation and inhibited platelet-platelet, platelet-monocyte, and platelet-neutrophil aggregation [25, 33, 50] and thus can protect against atherosclerosis and thrombosis [25]. Moreover, colchicine in vitro mitigated the mRNA and protein expression of TNF-α, IL-1β, IL-6, IL-18, MCP-1, ICAM-1 and VCAM-1 (Fig. 5) [5, 25, 34] and in vivo ameliorated their mRNA expression, circulating levels and protein expression in experimental animal studies [23, 26, 27]. MCP-1 and IL-1β deficient mice and blocking MCP-1 in rats showed impaired cerebral aneurysm formation and progression [14, 15]. The lack of IL-1β, MCP-1, and the inhibition of MCP-1 receptor CCR2 decreased atherosclerotic formation [16–18]. From these findings, it can be postulated that colchicine, by inhibiting SASP factors (Fig. 5) [5, 23, 25–27, 34], can alleviate the formation and progression of atherosclerosis and CAs [14–18].

MMPs released by senescent cells contribute to cardiovascular diseases via remodeling vascular tissue by degenerating extracellular matrix [10, 32]. In our study, colchicine inhibited mRNA and protein expression of MMP-2 (Fig. 5). Human CAs stained extensively for MMP-2 and MMP-9, while the circle of Willis arteries showed minimal or negative staining [51]. The protein expression of MMP-2 and MMP-9 was significantly upregulated in human CAs than in control arteries [51]. The mRNA expression of MMP-2 increased with the progression of CAs and the mRNA expression of MMP-9 was up-regulated after 3 months of CAs induction in rats [13]. Inhibiting MMP-2 and MMP-9 reduced the progression of CAs in rats [13]. MMP-2 deficiency significantly reduced atherosclerotic plaque lesion formation in mice [12]. MMPs can also modulate inflammation through their protease activity by post-translational processing of inflammatory cytokines such as TNF-α and IL1-β, chemokines like MCP-1, and cell adhesion molecules, namely ICAM-1 [11]. Colchicine reduced mRNA and protein expression of MMP2 in vitro [5], mitigated mRNA expression of MMP-3, MMP-9, and MMP-10 in aortas of mice [23] and abolished the relative mRNA expression of MMP-9 in infarct area of myocardium after myocardial infarction in mice [27]. Though colchicine alleviated the mRNA expression of many of the investigated SASP factors in oxidative stress-induced endothelial cells, colchicine alone increased the expression of SASP factors, including MMP-1, TIMP-1, and TIMP-2, which suggests a pro-senescent role of colchicine. TIMP-1 and TIMP-2 are extracellular inhibitors of MMPs, and by increasing their expression, colchicine could indirectly inhibit MMPs. These findings advocate that colchicine, by inhibiting MMP-2 mRNA and protein expression, can reduce tissue remodulation and thus can mitigate the formation and progression of atherosclerotic lesions and CAs (Fig. 5) [12, 13].

## Conclusion and future perspective

Endothelial cell senescence contributes to the progression of cardiovascular diseases. The current study shows that oxidative stress activates NF-kB, MAPKs, and mTOR pathways and induces premature senescence and SASP in endothelial cells. Colchicine suppressed oxidative stress-induced senescence and SASP in HUVECs by blocking the activation of the NF-kB and MAPKs pathways. In addition, to some extent, colchicine alone showed pro-senescent effects by increasing the expression of senescent markers, decreasing DNA repair proteins, and activating the mTOR pathway. These pro-senescent and cytotoxic effects of colchicine may hamper its use as a drug in the context of vascular diseases. Furthermore, more experiments would be needed to explore whether the lower concentration of colchicine can protect against oxidative stress-induced damage without its deleterious and cytotoxic effects. Because colchicine reduced the expression of MMPs and increased TIMPs expression, it can be an interesting drug to investigate its impact on metastasis in cancer cells.

## Methods

### Cell culture

HUVECs were commercially obtained from Promocell (Heidelberg, Germany) and maintained in the endothelial cell medium (C-22,010, Promocell, Heidelberg, Germany) supplemented with endothelial cell growth factors (C-39,215, Promocell, Heidelberg, Germany) at 37 °C in a 95% humidified atmosphere containing 5% CO_2_. Cells were seeded in the T75 adherent cell culture flask after thawing. When the cells came to 90% confluence, they were incubated with trypsin for 5 min at 37 °C, and then cells were used for experiments at passage 7. After 24 h, the medium was changed, and the new medium containing either H_2_O_2_ (300 µM), colchicine (50 nM), or H_2_O_2_ (300µM) combined with colchicine (50 nM) was added to the culture. Colchicine was purchased from Sigma-Aldrich (C3915).

### ROS Assay

ROS assay was performed using a Total Reactive Oxygen Species Assay Kit 520nM (Cat. Nr. 88-5930-74, Thermo Fisher Scientific) following the manufacturer’s instructions. Briefly, HUVECs were seeded in 96 well adhesion plates with a density of 2000 cells / cm2, then put into a 5% CO_2_, 95% humidity incubator for 24 h. The next day, the medium was changed with a new medium containing 1x ROS Assay Stain stock solution and incubated for 1 h. After that, either 300µM H_2_O_2_, 50 nM colchicine, or 300µM H_2_O_2_ combined with 50 nM colchicine were added to the medium already containing 1x ROS Assay Stain stock solution. The endothelial cell medium alone was added to the control cells. The measurements for ROS assay were performed with the Paradigm micro-plate reader after 2 h of treatments.

### Immunofluorescence staining

HUVECs were seeded in a 96-well plate (5000 cells/cm2). The medium was changed the next day with a new medium alone (for control) or a medium containing 300 µM H_2_O_2_, 50 nM colchicine, and a combination of 300 µM H_2_O_2_ and 50 nM colchicine. After two hours of treatment, immunofluorescence staining was performed. The cells were washed 3 times with PBS and fixed with 4% paraformaldehyde for 10 min, permeabilized with 0.2% Triton™ X-100 for 10 min, and blocked with 5% bovine serum albumin (BSA) for 1 h at RT. The cells were incubated overnight at 4° C with primary antibody 8-OHDG (1:500, Cat. No. BSS-BS-1278R, BIOSS, USA). The next day, the cells were washed three times with PBS and then labeled with a secondary antibody (1:1000, Alexa Fluor 488, ab150077, Abcam) for 1 h at RT. Nuclei were stained with DAPI (1ug/ml, 62,248, Thermo Fisher Scientific) for 10 min. The images were captured at 20x magnification under a Leica DMi8 Inverted Microscope and the compatible LAS-X Life Science Microscope Software Platform.

### β-Galactosidase (β-Gal) staining

β-Gal staining was performed with a β-Galactosidase Reporter Gene Staining Kit (Sigma-Aldrich, St. Louis, USA), following manufacturer instructions. The endothelial cells were treated with endothelial cell medium alone (control group) or endothelial cell medium supplemented with either 300 µM H_2_O_2_ or 50 nm colchicine or 300 µM H_2_O_2_ combined with 50 nM colchicine. After 24 h, the cells were fixed in the fixation buffer provided with the β-Galactosidase Reporter Gene Staining Kit. The fixed samples were stained at 37 °C for 7 h. After aspiration of the staining solution, the cells were overlaid with a 70% glycerol solution and stored at 4 °C. The images were taken with a Leica DMi8 Inverted Microscope and the compatible LAS-X Life Science Microscope Software Platform. Image J was used for the counting of stained cells. β-Gal staining was performed with three biological triplicates.

### Western blot

For protein analysis, HUVECs at P7 were treated with endothelial cell medium alone (Control group) or endothelial cell medium supplemented with either 300 µM H_2_O_2_, 50 nM colchicine, or 300 µM H_2_O_2_ combined with 50 nM colchicine in biological triplicates. After 24 h of treatment, the total protein was extracted using RIPA buffer, and protein concentration was determined using the DC Protein Assay Kit (500 − 0116, Bio-Rad, Hercules, CA, USA) following the manufacturer’s instructions and measured with the Paradigm micro-plate reader. Total protein (25 µg, in reducing conditions) was loaded on 12% sodium dodecyl sulfate-polyacrylamide gel and ran at 60 volts for 20 min, then continued with 110 Volts for 60 min, which was further transferred onto a polyvinylidene difluoride membrane at 250 mA for 120 min. The non-specific binding was blocked with 5% BSA dissolved in 0.05% TBST for 1 h. The membranes were incubated with primary antibodies (as reported in Supplementary Table 1) overnight at 4 °C on a shaking platform. The membranes were washed three times for 10 min with TBST and then incubated with secondary antibodies (as reported in Supplementary Table 1) for 1 h at room temperature. All primary antibodies were diluted in the blocking solution containing 5% BSA. The secondary antibodies were diluted in TBST. The densitometry of immunoblotted bands was calculated with Image J (Version 1.53t, National Institutes of Health, Bethesda, MD, USA). All experiments were performed in triplicates.

### Quantitative PCR

For quantitative PCR (qPCR) analysis, HUVECs at P7 were treated as described in the previous section. Total RNA was extracted using NucleoSpin RNA, Mini kit (740955.50, MACHEREY-NAGEL) following the manufacturer’s instructions. RNA (1.2 µg) was utilized to reverse transcript with M-MLV Reverse Transcriptase kit (M1701, Promega), Random Hexamer Primers (48,190,011, Thermo Fisher Scientific), and RiboLock RNase Inhibitor (EO0384, Thermo Fisher Scientific). qPCR was performed with AceQ SYBR qPCR Master Mix (Q111-03, Vayzme, Nanjing, China) on Bio-Rad CFX Connect Real-Time PCR System with an initial denaturation of 95 °C for 8 min, followed by 40 cycles of 95 °C for 15s, 58.9 °C for 30s, and 72 °C for 30s, followed by a melting curve. After normalizing to β-actin expression, the relative mRNA expressions were calculated using the comparative ΔCT method. The primer sequences used in experiments are reported in Supplementary Table 2.

### Statistical analysis

The data was analyzed by applying One-way ANOVA followed by Tukey’s test using Prism 9.5.0 (GraphPad Software Inc., San Diego, CA, USA). *P < 0.05 was considered significant.

### Limitations

Our study has some limitations. The study was performed using an in vitro model of primary endothelial cells. In the study, we used endothelial cells from the human umbilical vein and not from the artery. Also, the deleterious and cytotoxic effects of colchicine were not investigated. The expression of SASP factors was investigated at the mRNA level, which does not mean that the protein expression of these SASP factors will be similarly increased by oxidative stress and attenuated by colchicine. Therefore, the results should be interpreted carefully.

### Electronic supplementary material

Below is the link to the electronic supplementary material.

Supplementary Material 1: **Table 1**. Primary and Secondary antibodies. **Table 2**. Primer list

## Data Availability

All data generated or analyzed during this study are included in this published article [and its supplementary information files].
